# Role of the LytSR Two-Component Regulatory System in *Staphylococcus lugdunensis* Biofilm Formation and Pathogenesis

**DOI:** 10.3389/fmicb.2020.00039

**Published:** 2020-01-24

**Authors:** Sandrine Dahyot, Virginie Oxaran, Maïté Niepceron, Eddy Dupart, Stéphanie Legris, Laurie Destruel, Jennifer Didi, Thomas Clamens, Olivier Lesouhaitier, Yasmine Zerdoumi, Jean-Michel Flaman, Martine Pestel-Caron

**Affiliations:** ^1^Groupe de Recherche sur l’Adaptation Microbienne (GRAM 2.0), Department of Bacteriology, Rouen University Hospital, Normandie University, UNIROUEN, UNICAEN, Rouen, France; ^2^Department of Biological Sciences, Border Biomedical Research Center, University of Texas at El Paso, El Paso, TX, United States; ^3^Groupe de Recherche sur l’Adaptation Microbienne (GRAM 2.0), Normandie University, UNIROUEN, UNICAEN, Rouen, France; ^4^Laboratory of Microbiology Signals and Microenvironment (LMSM), Normandie University, UNIROUEN, Evreux, France; ^5^INSERM U1245, Normandy Centre for Genomic and Personalized Medicine, Rouen University Hospital, Normandie University, UNIROUEN, Rouen, France

**Keywords:** *S. lugdunensis*, biofilm, two-component system, LytSR, *Caenorhabditis elegans*, virulence, micro-array

## Abstract

*Staphylococcus lugdunensis* is a coagulase negative *Staphylococcus* recognized as a virulent pathogen. It is responsible for a wide variety of infections, some of which are associated with biofilm production, such as implanted medical device infections or endocarditis. However, little is known about *S. lugdunensis* regulation of virulence factor expression. Two-component regulatory systems (TCS) play a critical role in bacterial adaptation, survival, and virulence. Among them, LytSR is widely conserved but has variable roles in different organisms, all connected to metabolism or cell death and lysis occurring during biofilm development. Therefore, we investigated here the functions of LytSR in *S. lugdunensis* pathogenesis. Deletion of *lytSR* in *S. lugdunensis* DSM 4804 strain did not alter either susceptibility to Triton X-100 induced autolysis or death induced by antibiotics targeting cell wall synthesis. Interestingly, Δ*lytSR* biofilm was characterized by a lower biomass, a lack of tower structures, and a higher rate of dead cells compared to the wild-type strain. Virulence toward *Caenorhabditis elegans* using a slow-killing assay was significantly reduced for the mutant compared to the wild-type strain. By contrast, the deletion of *lytSR* had no effect on the cytotoxicity of *S. lugdunensis* toward the human keratinocyte cell line HaCaT. Transcriptional analyses conducted at mid- and late-exponential phases showed that *lytSR* deletion affected the expression of 286 genes. Most of them were involved in basic functions such as the metabolism of amino acids, carbohydrates, and nucleotides. Furthermore, LytSR appeared to be involved in the regulation of genes encoding known or putative virulence and colonization factors, including the fibrinogen-binding protein Fbl, the major autolysin AtlL, and the type VII secretion system. Overall, our data suggest that the LytSR TCS is implicated in *S. lugdunensis* pathogenesis, through its involvement in biofilm formation and potentially by the control of genes encoding putative virulence factors.

## Introduction

*Staphylococcus lugdunensis* is a member of the coagulase-negative staphylococci (CoNS) family. This bacterium belongs to the human skin flora; van der Mee-Marquet et al. found, for example, carriage of inguinal *S. lugdunensis* in 22% of 140 incoming patients (van der Mee-Marquet et al., 2003). However, its pathogenicity is closer to that of *Staphylococcus aureus* than that of other CoNS in terms of tissue destruction ability and clinical course (Frank et al., 2008; Heilmann et al., 2019). In particular, *S. lugdunensis* native valve endocarditis can be particularly aggressive and destructive, often requiring surgery (Anguera et al., 2005). It can also cause a wide range of infections such as abscesses and wound infections (Böcher et al., 2009; Heldt Manica and Cohen, 2017), bone and joint infections (Argemi et al., 2017b), and infections associated with catheters or implanted medical devices (Nesher et al., 2017). Only a few virulence factors have been identified so far to explain such pathogenicity (Heilbronner et al., 2011; Argemi et al., 2017a). Similar to other staphylococci, the pathogeny of *S. lugdunensis* appears in many infections to be related to biofilm formation within host tissues or indwelling medical devices (Frank and Patel, 2007; Argemi et al., 2017a). The resulting infections are usually difficult to treat because biofilm protects bacteria from both the host’s immune system and the antimicrobial therapies (Stewart and Costerton, 2001; Lebeaux et al., 2014).

In staphylococci, biofilm formation appears to depend mainly on proteins (as in *S. aureus*), or on the exopolysaccharide poly-*N*-acetylglucosamine (PNAG), also termed polysaccharide intercellular adhesion (PIA), as described for *Staphylococcus epidermidis* (Otto, 2018). PIA is encoded by the *icaADBC* operon composed of four genes whose expression is regulated by the transcriptional repressor IcaR. Despite the identification of an *icaADBC locus*, PIA is not a major component of the *S. lugdunensis* biofilm matrix (Frank and Patel, 2007). Instead, biofilm consists of extracellular factors, which are mainly proteinaceous. However, not all of these proteins have been identified yet. Among the identified surface proteins that are involved in *S. lugdunensis* biofilm formation, the iron-regulated surface determinant IsdC under iron-limited conditions (Missineo et al., 2014) and the major autolysin AtlL (Gibert et al., 2014; Hussain et al., 2015) have been demonstrated to be important actors.

Despite their clinical significance, the production and regulation of staphylococcal biofilm remain poorly defined. It is assumed that environmental signals can influence the polysaccharide or proteinaceous composition of the biofilm matrix (Vergara-Irigaray et al., 2009). Indeed, some isolates of *S. aureus* and *S. epidermidis* are able to form biofilm without PIA (Rohde et al., 2007; Arciola et al., 2015). The ability of bacteria to adapt to environment is mediated by complex regulatory networks, including two-component signal transduction systems (TCSs), which allow a pathogen to adapt its gene expression in response to environmental stimuli (Beier and Gross, 2006; Haag and Bagnoli, 2017). Some of these TCSs are involved in controlling staphylococcal biofilm formation, such as *agr* (Kavanaugh and Horswill, 2016), *arlRS* (Burgui et al., 2018), and *lytSR* (Sharma-Kuinkel et al., 2009). Interestingly, the LytSR TCS plays an important role in biofilm development in *S. aureus* (Sharma-Kuinkel et al., 2009) as well as in *S. epidermidis* (Zhu et al., 2010) through an *ica*-independent mechanism.

The LytSR system was first identified in *S. aureus,* where it was shown to affect murein hydrolase activity and autolysis (Brunskill and Bayles, 1996a). Further characterization demonstrated that LytSR positively regulates the expression of the immediately downstream *lrgAB* operon (Brunskill and Bayles, 1996b), which, along with the *cidABC* operon, is implicated in the control of programmed cell death and lysis during biofilm development (Bayles, 2007; Rice et al., 2007; Sadykov and Bayles, 2012). Indeed, *cidA* gene encodes a holin-like protein that is an effector of extracellular murein hydrolase activity, cell lysis, and DNA release, while *lrgA* encodes an anti-holin-like protein, which is an inhibitor of these processes (Rice et al., 2007). Thus, the subpopulation of dead bacteria that lyses within the biofilm releases extracellular DNA (eDNA), which plays a critical role in intercellular adhesion and biofilm stability (Bayles, 2007). As *lrg* and *cid* operons are involved in autolysis and cell death (van den Esker et al., 2017), their transcription is subject to complex control. They are under the control of two overlapping regulatory networks, one in response to the metabolism of carbohydrates (Yang et al., 2005; Patel and Golemi-Kotra, 2015) and the other in response to changes in membrane potential (Patton et al., 2006).

Given the critical role of LytSR in the control of lysis and cell death in the biofilm of *S. aureus* and *S. epidermidis*, it seemed interesting to explore its functions in the particularly virulent CoNS, *S. lugdunensis*. In the present study, we constructed a DSM 4804 Δ*lytSR* strain and investigated the effects of this deletion on bacterial autolysis, biofilm formation, and *in vivo* virulence. Furthermore, we set out to determine the full extent of the *S. lugdunensis* LytSR regulon by transcriptional profile analysis.

## Materials and Methods

### Bacterial Strains and Growth Conditions

The bacterial strains and plasmids used in this study are listed in Table 1. Unless otherwise stated, *S. lugdunensis* strains were grown in trypticase soy broth (TSB; Bio-Rad, Marnes-la-Coquette, France), while *Escherichia coli* TOP10 strain (Invitrogen, Thermo Fisher Scientific, Massachusetts, USA) was cultured in Luria-Bertani medium (Bio-Rad) at 37°C under aerobic conditions. When necessary, antibiotics were used in the following concentrations: ampicillin, 100 μg/ml; chloramphenicol, 50 μg/ml; and erythromycin, 2.5 μg/ml (Sigma-Aldrich, St. Louis, USA).

**Table 1:** Bacterial strains and plasmids.

Strain or plasmid	Description	Reference
**Strains**
***S. lugdunensis***		
DSM 4804 (WT)	Clinical axillary lymph node isolate	Freney et al. (1988)
WT Δ*lytSR*	*lytSR* mutant	This study
WT Δ*lytSR* (pCU1)	*lytSR* mutant containing the empty pCU1 vector	This study
WT Δ*lytSR* (pCU1:*lytSR*)	*lytSR* complementary strain	This study
WT Δ*atlL*	*atlL* mutant resistant to autolysis	Gibert et al. (2014)
***E. coli***		
TOP10	Host strain for construction of recombinant plasmid	Invitrogen
**Plasmids**
pMAD	Temperature-sensitive shuttle vector; Amp^R^, Ery^R^	Arnaud et al. (2004)
pCU1	Shuttle vector; Amp^R^, Cm^R^	Augustin et al. (1992)

*Amp^R^, ampicillin resistance; Ery^R^, erythromycin resistance; Cm^R^, chloramphenicol resistance*.

### DNA Extraction and Polymerase Chain Reaction Amplification

Isolates were grown overnight at 37°C on tryptic soy agar (TSA; Bio-Rad) supplemented with 5% horse blood. DNA was extracted using the InstaGene Matrix kit (Bio-Rad) according to the manufacturer’s instructions. PCRs were performed using a Veriti Thermal Cycler (Applied Biosystems, Foster City, CA, USA) in a final volume of 25 μl containing 12.5 μl GoTaq G2 Green Master Mix (Promega, Charbonnières-les-Bains, France), 0.50 μM of each primer, and 5 μl of DNA. When necessary, PCR products were purified and verified by sequencing by Eurofins GATC Biotech SARL (Konstanz, Germany).

### Construction of the *S. lugdunensis* DSMΔ*lytSR* Mutant and Complementation Strains

The *lytSR* deletion mutant was constructed by homologous recombination using pMAD plasmid (Arnaud et al., 2004). The fragment containing the upstream region of *lytS* (with respect to the *lytS* translation initiation site) and the downstream region of *lytR* (with respect to the *lytR* stop codon) was amplified by a two-step overlap PCR reaction using the oligonucleotide pairs lytS_EcoRI_F/ lytS_R and lytR_ F/lytR_BamHI_R (Table 2). The purified PCR product was digested with EcoRI and BamHI restriction enzymes (New England BioLabs, Évry, France) and ligated into the pMAD vector with T4 DNA ligase (New England BioLabs) following the manufacturer’s instructions and then cloned into *E. coli* TOP10. Clones containing the resulting recombinant pMADΔ*lytSR* vector were selected on TSA supplemented with ampicillin. The recombinant plasmid pMADΔ*lytSR* was extracted (NucleoBond Xtra Midi endotoxin free, Macherey Nagel, Hœrdt, France) and used to transform the wild-type (WT) *S. lugdunensis* DSM 4804 strain (Freney et al., 1988) by protoplast formation as previously described (Szabados et al., 2011; Marlinghaus et al., 2012). After incubation at 30°C for 5 days, clones containing the plasmid were selected, and the double-crossover event was performed by a shift of temperature to 43°C and in the presence of erythromycin (Arnaud et al., 2004). The loss of the plasmid was obtained after successive shifts between 30 and 43°C and in the absence of antibiotic. Successful deletion of the *lytSR* operon *via* homologous recombination and loss of the plasmid was verified by PCR and sequencing.

**Table 2:** Primers.

Primer	Sequence (5′-3′)	Restriction enzyme	Reference
**lytSR deletion**
lytS_EcoRI_F	AGGCTGAATTCATAATGAACCCACGATATTTAATGCTAG	EcoRI	This study
lytS_R	CGTGTGTTAGATTTATGCCATTGTGCCATACTCCCAAAAAAATATT		This study
lytR_ F	TGGGAGTATGGCACAATGAGCATAAATCTAACACACGAATCAAATG		This study
lytR_BamHI_R	ATTGGATCCCTGGCATTGGAAACGGTATAAAAC	BamHI	This study
			
**lytSR complementation**	
5′_lytS	AGGCTGAATTCCAAACTGAGATGAATGATTGTATATTGAAAA	EcoRI	This study
3′_lytR	ATTGGATCCACATCCCCGACATACAAAAAACAC	BamHI	This study
			
**(q)RT-PCR analyses**	
lytS1_F	CCAGTGCCTGTTTCAGAGTTG		This study
lytS1_R	CACGACGATGCGATTCAATTAAC		This study
lytR1_F	TGCCATTATTGACGGTTACGG		This study
lytR2_R	AAACACGCATACGAAGCAAAC		This study
lytS2_F	GATGCGATTCAATTAACTGTA		This study
lytR2_R	AAAATAATGTAAGGTGCATGT		This study
lrgA_F	ACGCTGTACCAACACTTTCAAC		This study
lrgA_R	CCAATGCCAGCTTCAGTAATAGG		This study
lrgB_F	CCAACGATAACGACTGCAATACTG		This study
lrgB_R	TTAGGCACAAGCGGACATACAC		This study
cidA_F	TAGCAGGCAGTATTGTAGGC		This study
cidA_R	ACCCGTCTTTCACCCATTG		This study
16S_F (Q3)	GAGGAAGGIGIGGAIGACGT		Tseng et al. (2003)
16S_R (Q4)	AGICCCGIGAACGTATTCAC		Tseng et al. (2003)

To construct the complemented strain, the *lytSR* operon including its predicted promoter and ribosome binding site was amplified by PCR using primers 5′_lytS and 3′_lytR (282 bp upstream *lytS* and 441 bp downstream *lytR*; Table 2) and was ligated into the pCU1 plasmid (Augustin et al., 1992) at the EcoRI/BamHI restriction sites and subcloned into *E. coli* TOP10. After verification by PCR and sequencing of the vector pCU1: *lytSR*, this vector and the empty vector pCU1 were transformed into *S. lugdunensis* DSMΔ*lytSR* by protoplast formation.

### Triton X-100-Induced Autolysis

Triton X-100-induced autolysis assay was performed as previously described (Brunskill and Bayles, 1996a). Briefly, cells were grown in TSB containing 1 M NaCl to mid-exponential phase (OD_600 nm_ = 0.7), and 50 ml was pelleted by centrifugation. Cells were washed twice with 50 ml of ice-cold sterile water and resuspended in 50 ml of 0.05 M Tris-HCl (pH 7.2) containing 0.05% (vol/vol) Triton X-100. Cells were then incubated at 37°C under shaking (180 rpm). OD_600 nm_ was measured at 15-min intervals for 1 h and then at 30-min intervals for 2 h to evaluate autolysis. All experiments were conducted using at least three biological replicates.

### Antibiotic Time-Kill Assays

For time-kill kinetic assays, bacteria were subcultured in Mueller-Hinton broth (MHB) to early-exponential phase (3 h), diluted to approximately 5 × 10^5^ CFU/ml in MHB containing antimicrobial agents, and incubated for 24 h at 37°C (National Committee for Clinical Laboratory Standards, 1999). Penicillin G, vancomycin, and teicoplanin were added to a final concentration equivalent to 10 times their MICs (i.e. final concentration of 0.64 mg/L for penicillin G, 10 mg/L for vancomycin, and 2.5 mg/L for teicoplanin). Bacterial counts were performed just before, at 6 and 24 h after antibiotic addition. A 1 ml aliquot was taken and serially diluted in 0.9% sodium chloride, and viable cells were quantified by plating dilutions on TSA. Tolerance was defined as a <3-log_10_ reduction of the bacterial count after 24 h according to CLSI guidelines (National Committee for Clinical Laboratory Standards, 1999). We also carried out studies, in which bacteria approaching the stationary phase (10 h) were treated with penicillin G (final concentration 0.64 mg/L). Cell viability was assessed at 2, 4, 6, and 8 h after antibiotic addition. All experiments were repeated at least three times.

### Biofilm Assays and Confocal Laser Scanning Microscopy

Overnight cultures of *S. lugdunensis* WT and mutant strains grown in TSB supplemented with 1% glucose (TSB_1%glc_) were adjusted to an OD_600nm_ of 0.01 in TSB_1%glc_. Cultures were distributed in triplicate into six-well flat-bottom plate. After 24 h incubation at 37°C, cells were washed gently two times with sterile distilled water and stained with SYTO®9 and propidium iodide (PI) (Live/Dead kit, Thermo Fisher Scientific). The architecture of biofilms was observed with a confocal laser scanning microscope (Leica TCS SP2). CLSM z-stack processing was performed using Leica Confocal Software. Measurements of the biofilms produced were performed using IMARIS software (Bitplane, Belfast, Ireland), calculating the biomass and the percentage of cells labeled by SYTO®9 and PI. Each confocal experiment was repeated a minimum of three times. Statistical significance was calculated by the chi-squared test with Yates continuity correction; *p* < 0.05 was considered as significant.

### *Caenorhabditis elegans* Virulence Assays

The *Caenorhabditis elegans* WT Bristol strain N2 was obtained from the *Caenorhabditis* Genetics Center (Minneapolis, MN, USA). *C. elegans* worms were maintained under standard culturing conditions at 23°C on nematode growth medium (NGM; 3 g NaCl, 2.5 g peptone, 17 g agar, 5 mg cholesterol, 1 ml 1 M CaCl_2_, 1 ml 1 M MgSO_4_, 25 ml 1 M KH_2_PO_4_, H_2_O to 1 L) agar plates with *Escherichia coli* OP50 as a food source. Synchronous cultures of worms were generated as previously described (Blier et al., 2011) and used for both slow-killing assay and liquid-killing assay.

Bacterial lawns used for *C. elegans* survival slow-killing assays were prepared by spreading 50 μl of WT or ∆*lytSR* strains on 35 mm NGM conditioned Petri dishes supplemented with both 0.05 mg/ml of 5-fluoro-2′-deoxyuridine and 5 μmol/L of tryptophan. Plates were incubated overnight at 37°C and then placed at room temperature for 4 h. Fifteen to twenty L4 (48-h old) synchronized nematodes were harvested with M9 solution (3 g KH_2_PO_4_, 6 g NaHPO_4_, 5 g NaCl, 1 ml 1 M MgSO_4_, H_2_O to 1 L), placed on 35 mm assay Petri dishes and incubated at 23°C. *C. elegans* survival was scored at 1, 24 h and each subsequent day until death, using an Axiovert S100 optical microscope (Zeiss, Oberkochen, Germany) equipped with a Nikon digital Camera DXM 1200F (Nikon Instruments, Melville, NY, USA). The nematodes were considered dead when they remained static without grinder movements for 20 s or did not respond to light flashes. The results are expressed as the percentage of living nematodes and correspond to the mean of two independent assays. Nematode survival was calculated by the Kaplan-Meier method, and differences in survival kinetics were tested for significance by using the log rank test (GraphPad Prism version 4.0; GraphPad Software, San Diego, CA, USA).

A liquid killing assay (Chua et al., 2014) was also performed in order to evaluate the toxicity of bacterial released factors toward *C. elegans*. Volumes (80 μl) of 5-h old bacterial cultures were distributed onto microtiter plate wells. Immediately after, 20 μl of M9 solution containing 15–20 L4 (48 h old) synchronized worms was added to bacteria in each well and incubated at 23°C. Exact worm number was immediately determined (*t* = 0 h), and worm survival was scored at different time points (24, 48, and 120 h) using an Axiovert S100 optical microscope (Zeiss). Each value reported for the assays is the mean measurement for six replicates of six independent preparations. The nonparametric Mann-Whitney *U* test was used to compare the means within the sets of experiments, using Past 3.x software.

### HaCaT Cell Cytotoxicity Tests

The cytotoxicity activity of bacteria was studied using the human keratinocyte cell line HaCaT (Eppelheim, Germany) as previously described (N’Diaye et al., 2016). Briefly, HaCaT cells were grown at 37°C under 5% CO_2_ atmosphere, in Dulbecco’s modified Eagle’s medium (DMEM, Lonza, Levallois-Perret, France) supplemented with 10% fetal calf serum and 1% antibiotic cocktail (HyClone Thermo Scientific, Illkirch, France). Cells were used between passages 41 and 65. One day before use, HaCaT cells were starved of antibiotic and fetal calf serum. Cells were incubated for 24 h with bacteria at a multiplicity of infection (MOI) of 10:1. Bacterial cytotoxicity was determined by measurement of lactate dehydrogenase (LDH) release by HaCaT cells. LDH is a stable cytosolic enzyme that diffuses into the culture medium upon cell lysis and was measured using a Cytotox 96 assay (Promega). Results are the mean of three independent experiments each done in independent triplicate measurement.

### RNA Extraction and Quantitative Real-Time Polymerase Chain Reaction

Cells were grown in TSB at 37°C under shaking conditions (150 rpm). One milliliter was centrifuged (5 min at 8,000 ×*g*, 4°C) at different time points (4, 6, 8, and 24 h). RNA was extracted using the NucleoSpin® RNA kit (Macherey Nagel) according to the manufacturer’s instructions. cDNA synthesis was then performed using the OmniScript® RT kit (Qiagen, Hilden, Germany), as previously described (Dahyot et al., 2019). To validate the differential gene expression obtained by microarray, qRT-PCR was carried out using a CFX96 real-time PCR detection system (Bio-Rad) with SYBR® Green PCR Master Mix (Bio-Rad), 1 μl of diluted cDNA template, and primers listed in Table 2 in 20 μl PCR mixtures. Cycling conditions were one initial denaturation step at 95°C for 10 min, followed by 40 cycles of amplification including denaturation at 95°C for 30 s, annealing at Tm depending on the primers for 30 s, and extension at 72°C for 30 s. Relative expression of genes was quantified using the gene expression analysis module of CFX Manager™ software, with 16S rRNA gene as reference to normalize the results. All experiments were performed in at least three independent RNA preparations, and results are presented as the mean ± the standard error of the mean.

### RNA Extraction and Sample Preparation for Microarray

To decipher the mechanisms affected by the deletion of *lytRS* genes, the transcriptome of Δ*lytSR* and WT strains was compared by DNA microarray at 6 and 8 h of growth. *S. lugdunensis* was grown in TSB, and cells were collected at 6 and 8 h of growth. Total RNA was extracted using the RNeasy Mini kit (Qiagen) following the manufacturer’s instructions. Two DNase treatments were successively performed using DNase I (Sigma-Aldrich) and Turbo DNA free (Thermo Fisher Scientific). The quality of RNA was evaluated using the RNA 6000 Nano with a Bioanalyzer (Agilent, Les Ulis, France). The preparation of samples and array processing were performed using the Two-color Microbased Exon Analysis kit (Agilent) following the manufacturer’s instructions. Briefly, cRNA was labeled with either Cy3 or Cy5 before being quantified and co-hybridized on the microarray. The custom-made *S. lugdunensis* GeneChips (Agilent) used in this study were generated based on DNA sequences from two publicly available genomes, *S. lugdunensis* N920143 and HKU09–01 strains (Tse et al., 2010; Heilbronner et al., 2011). Then, the microarray was washed and scanned. Genes with significantly different expression (*p* ≤ 0.05) were selected based on at least a two-fold change in expression level. The genes identified were grouped following the classification established in the KEGG pathway database^1^.

## Results

### *In silico* and Transcription Analyses of the *lytSR locus*

We first amplified and sequenced the *lytSR* region of the *S. lugdunensis* DSM 4804 strain. Sequence analysis revealed a 1755-nucleotide *lytS* open reading frame (ORF) immediately followed by a 762-nucleotide *lytR* ORF. BLAST searches indicated 67% homology with the *lytSR* operon of the *S. aureus* NCTC5661 strain and 69% with the *S. epidermidis* ATCC 12228 strain (Zhang et al., 2003). The transcription start site was tentatively located 63 bp upstream of the *lytS* gene, preceded by a canonical −10 element (TGTTAAAAT) and near-canonical −35 element (TTAACA). A consensus ribosome-binding site (GGGAG) was found 9 bp upstream of the *lytS* ATG start codon.

The predicted amino acid sequence of *lytS* gene product (LytS) contains 584 amino acids corresponding to a 64.83 kDa protein. This protein shares 77 and 71% amino acid sequence identity with *S. aureus* and *S. epidermidis,* respectively. *lytR* gene encodes a putative 253 amino acid protein (29.4 kDa), which shares 64 and 63% amino acid identity with the LytR proteins of *S. aureus* and *S. epidermidis*, respectively. The predicted LytS and LytR proteins share putative conserved domains with members of a two-component regulatory system, namely a sensor histidine kinase protein and a response regulator transcription factor, respectively. Sequence analysis revealed at 510 bp downstream *lytR* the presence of *lrgA* and *lrgB* genes predicted to encode two anti-holin-like proteins.

As it has been shown that *lytSR* and *lrgAB* are temporally regulated in *S. aureus* (Groicher et al., 2000), the expression of these genes was analyzed by qRT-PCR assays at various culture times of *S. lugdunensis* DSM 4804. Transcription of *lytS* and *lytR* genes appeared maximal at 6 h growth (mid-exponential phase), whereas that of *lrgA* and *lrgB* genes reached its maximum at 8 h (late-exponential phase) (data not shown). A fragment overlapping *lytS* and *lytR* genes was obtained by RT-PCR analysis, indicating that these genes are co-transcribed (data not shown).

### Construction of the *S. lugdunensis* Δ*lytSR* Strain and the Complemented Strains

**Figure 1:**
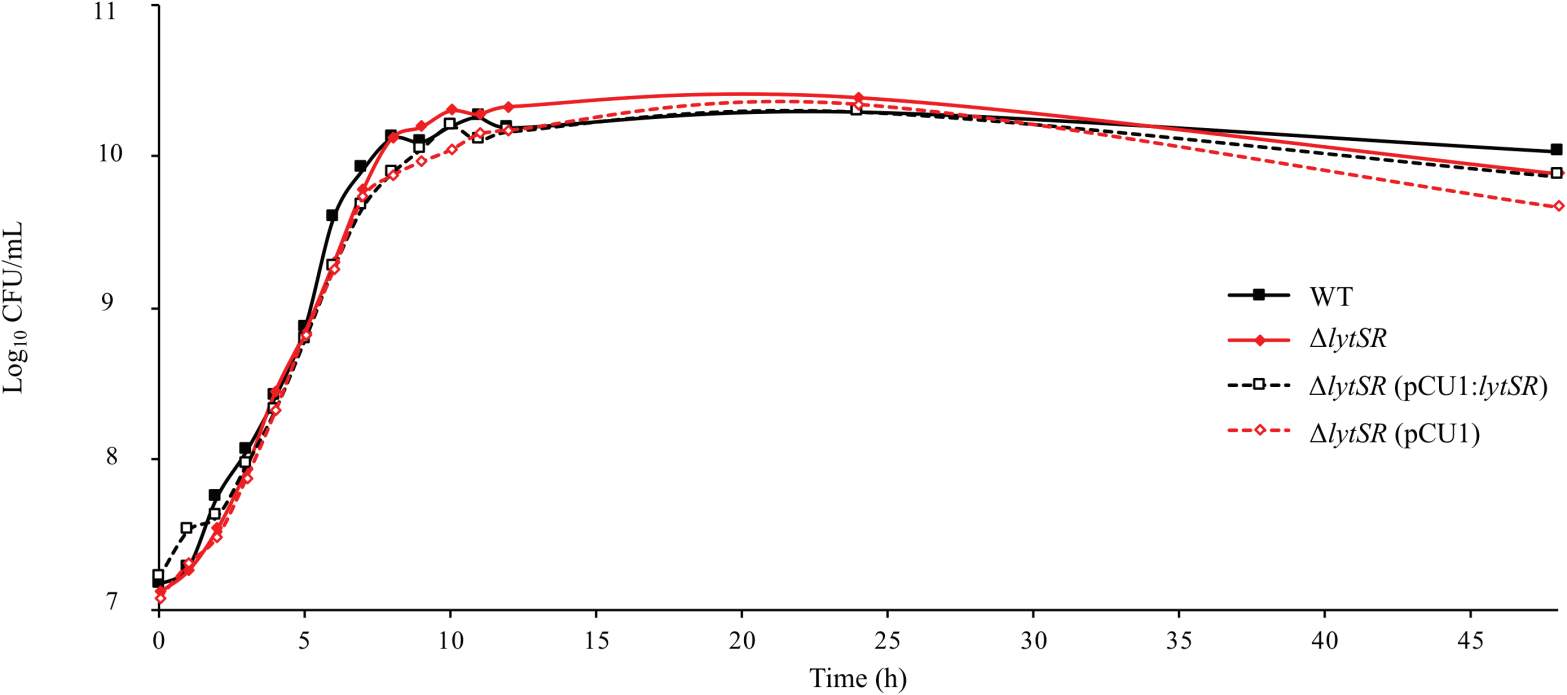
Growth curves of *S. lugdunensis* WT, Δ*lytSR,* and complemented strains. Bacterial cultures were grown in TSB at 37°C. Growth was monitored by measuring OD_600 nm_, and viable cell counts were determined by plating diluted aliquots on TSA. Datum points represent the means of three independent experiments for WT and mutant strains.

To investigate the roles of LytSR in *S. lugdunensis*, a Δ*lytSR* strain was constructed by homologous recombination. We verified by PCR that the Δ*lytSR* strain presented the 1,200-bp deletion at *lytSR locus*. In order to exclude a polar effect of this deletion, we carried out complementation of the Δ*lytSR* strain using pCU1:*lytSR* plasmid. The Δ*lytSR* strain containing pCU1 plasmid was used as a negative control. All four strains showed a similar growth rate in TSB medium with no difference in cell viability (i.e. no difference in CFU/ml counts when incubated 48 h at 37°C; Figure 1).

### Implication of LytSR in Autolysis

**Figure 2:**
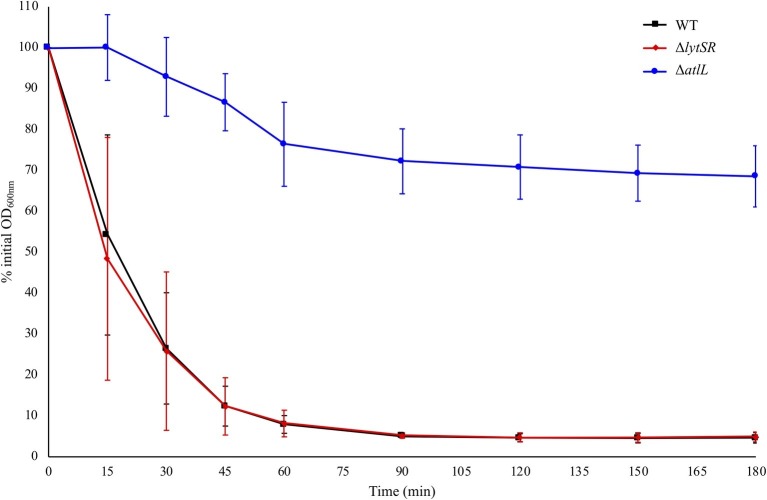
Triton X-100 autolysis assays of *S. lugdunensis* WT and derivative (Δ*lytSR* and Δ*atlL*) strains. Bacterial cells were collected from early-exponential growth (OD_600 nm_ = 0.7) and resuspended in an equal volume of buffer containing 0.05% Triton X-100. The rate of autolysis was monitored at OD_600 nm_. The *S. lugdunensis atlL* deletion mutant was used as a control for resistance to autolysis. Error bars represent the SD of three independent experiments.

To determine the contribution of LytSR in the regulation of autolysis, Triton X-100-induced autolysis of *S. lugdunensis* WT and Δ*lytSR* strains was measured. Both strains showed a similar trend of autolysis, while the *S. lugdunensis* Δ*atlL* strain used as a negative control (Gibert et al., 2014) showed increased resistance to autolysis (Figure 2).

### Impact of *lytSR* Deletion on Antibiotic-Induced Killing

**Figure 3:**
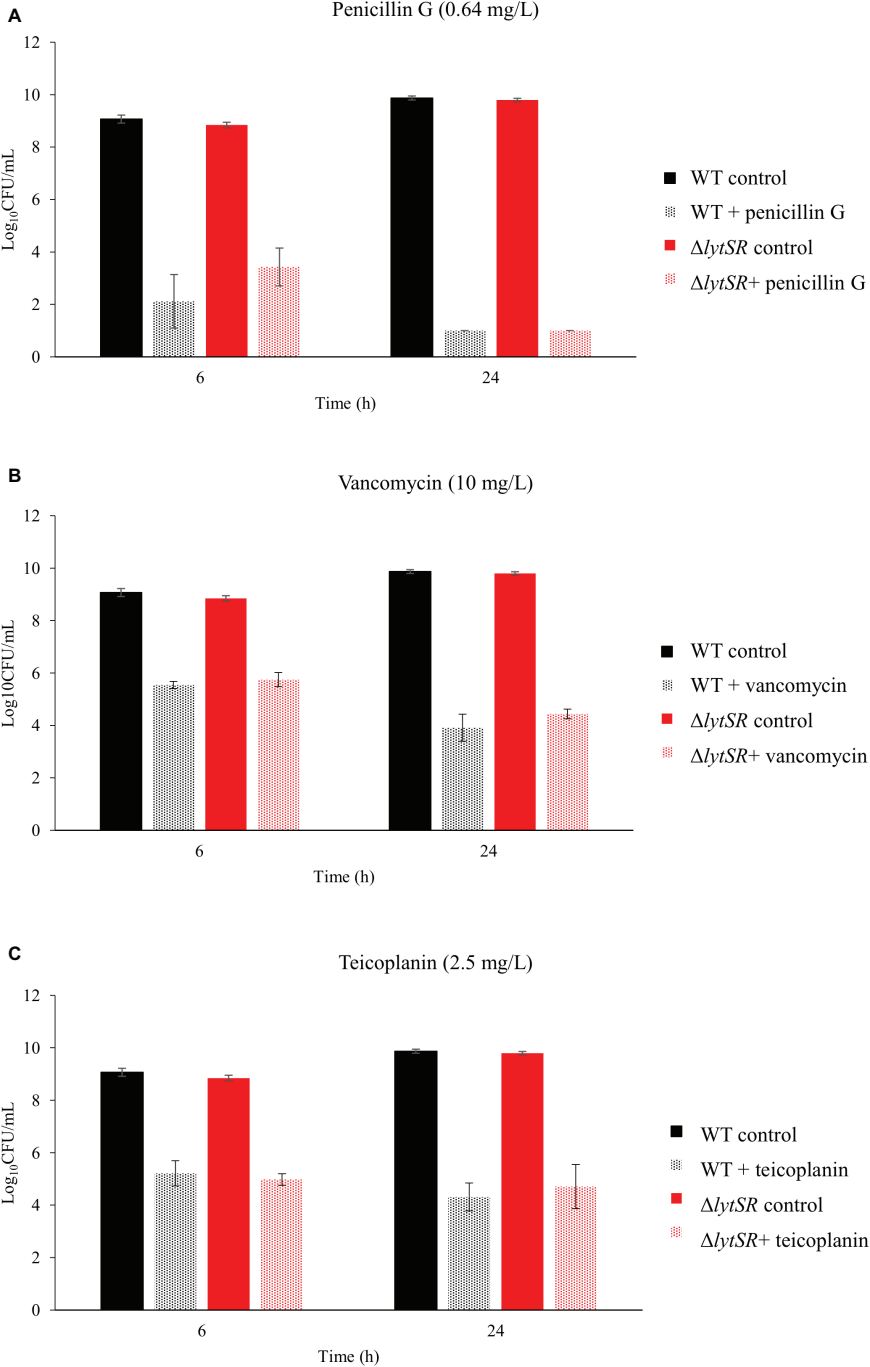
Time-kill kinetics of **(A)** penicillin G, **(B)** vancomycin, and **(C)** teicoplanin against *S. lugdunensis* WT and Δ*lytSR* strains. Antibiotics (10 times the MICs) were added to early-exponential growth cultures of the strains. Viable bacterial counts were determined after 6 and 24 h of antibiotic exposure by plating diluted aliquots on TSA. Histograms are expressed as mean ± SD of three independent experiments.

The possible role of LytSR in antibiotic-induced cell death was assessed by time-kill kinetic assays. A β-lactam (penicillin G, Figure 3A) and two glycopeptides (vancomycin, Figure 3B and teicoplanin, Figure 3C), which interfere with peptidoglycan biosynthesis, were used. The MICs of these antibiotics were the same for WT and mutant strains (data not shown). Penicillin G or glycopeptides were added to early-exponential growth cultures at a lytic concentration (10 times their MICs), and the rate of living cells over time was determined. The decrease in bacterial counts was not significantly different between the WT and the mutant strains, exposed to either penicillin G or glycopeptides, for 6 and 24 h (Figure 3 and Table 3). Penicillin G was bactericidal with a 5-log CFU/ml reduction after 24 h exposure for both strains. On the contrary, the WT strain was tolerant to glycopeptides, a phenomenon also observed for the Δ*lytSR* strain (i.e. ΔlogCFU/ml < 3 at 24 h). Furthermore, *lytSR* deletion did not promote penicillin G-induced killing of cells entering stationary phase of growth (data not shown).

**Table 3:** Effect of *lytSR* deletion on antibiotic sensitivity.

Mean ± SD ΔlogCFU/ml						
	Penicillin G		Vancomycin		Teicoplanin	
	6 h	24 h	6 h	24 h	6 h	24 h
WT	−4.36 ± 0.98	−5.48 ± 0.04	−0.92 ± 0.14	−2.55 ± 0.48	−1.23 ± 0.45	−2.13 ± 0.43
∆*lytSR*	−2.88 ± 0.73	−5.31 ± 0.04	−0.60 ± 0.18	−1.91 ± 0.10	−1.39 ± 0.24	−1.66 ± 0.76

*Penicillin G or two glycopeptides, vancomycin, and teicoplanin, were added at a concentration of 10 times the MICs to early-exponential growth cultures of S. lugdunensis WT and ΔlytSR strains. Reduction of bacterial counts was determined after 6 and 24 h by plating diluted aliquots on TSA. Data are means ± SD of three independent experiments*.

### Impact of *lytSR* Deletion on Biofilm Formation

**Figure 4:**
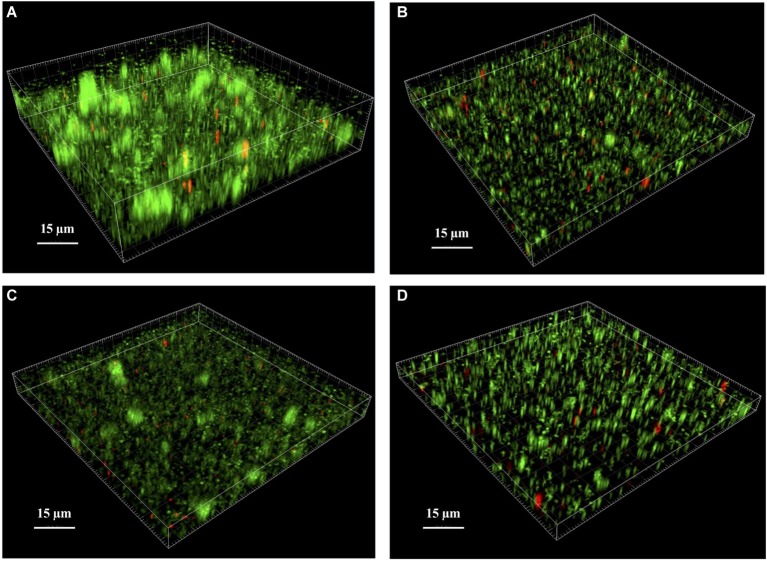
Analysis of *S. lugdunensis* biofilm by confocal laser scanning microscopy (CLSM). The 24 h mature biofilms of **(A)** *S. lugdunensis* WT, **(B)** Δ*lytSR*, **(C)** Δ*lytSR* (pCU1:*lytSR*), and **(D)** Δ*lytSR* (pCU1) were visualized after Live/Dead staining under CLSM. Live cells stained with SYTO®9 appear in green, while dead cells stained with propidium iodide are in red. Three-dimensional structural images were reconstructed, and the amount of fluorescence of viable and dead cells was determined using Imaris software. The figures represent one of three independent experiments.

We examined the biofilm architecture and the cell viability of WT, mutant, and complemented strains using CLSM. With the Live/Dead viability staining method, bacteria with intact cell membranes were stained in green, whereas bacteria with damaged membranes were stained in red. First, we observed that the mature biofilm of the WT strain contained typical tower structures (Figure 4A) that were not observed in the biofilm of either the Δ*lytSR* strain (Figure 4B) or the Δ*lytSR* strain containing empty pCU1 (Figure 4D). Complementation with *lytSR* partially restored these structures (Figure 4C). The total biomass of the biofilm was two-fold lower for the mutant strain than the WT strain (Supplementary Table S1). Moreover, the percentage of dead cells was significantly increased in the biofilm of the Δ*lytSR* strain (5.7%) compared to the parental strain (1.1%; *p* < 0.05, chi-squared test; Supplementary Table S1). Both complemented strains exhibited higher levels of dead cell probably because of antibiotic selection pressure. However, the Δ*lytSR* strain complemented with *lytSR* exhibited a significantly lower percentage of dead cells (10.4%) than the mutant complemented with the empty pCU1vector (15.2%; *p* < 0.05, chi-squared test; Supplementary Table S1).

### Impact of LytSR on Virulence Toward *Caenorhabditis elegans*

**Figure 5:**
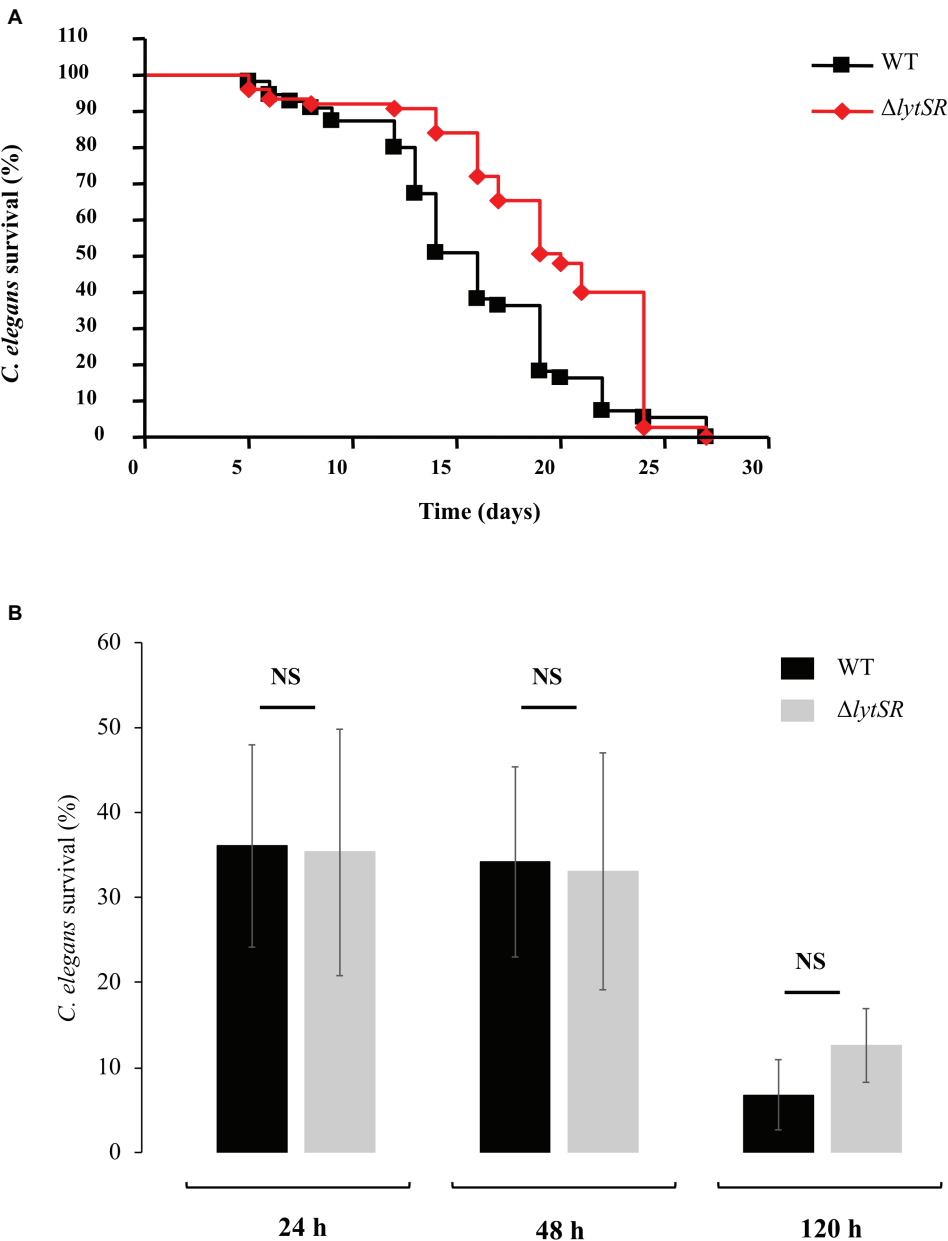
*S. lugdunensis* virulence toward *Caenorhabditis elegans* worms. **(A)** Kinetics of survival of *C. elegans* fed with WT and Δ*lytSR* strains in solid killing assay. Each value is the mean of measurement of eight replicates from two independent preparations. Nematode survival was calculated by the Kaplan-Meier method, and survival differences were tested for significance using the log rank test. The survival kinetics of *C. elegans* was significantly increased in the presence of Δ*lytSR* strain (*p* < 0.0001). **(B)** Liquid killing assay of *C. elegans* exposed to the supernatant of WT and Δ*lytSR* strains. Nematode survival was evaluated after 24, 48, and 120 h of exposure. Each value is the mean ± SEM of measurement of eight replicates from six independent preparations. The nonparametric Mann-Whitney *U* test was used to compare the means within the same set of experiments. NS, not significant.

We assessed the effect of the *lytSR* deletion on pathogenesis of *S. lugdunensis* using the *C. elegans* infection model. Slow-killing test showed that when nematodes were in contact with the WT strain as the sole source of food, it took 15 days to kill 50% of *C. elegans* (Figure 5A). Interestingly, the survival of *C. elegans* in the presence of the Δ*lytSR* strain was significantly increased (*p* < 0.0001), with 19 days required for 50% of the nematodes to die (Figure 5A). By contrast, no difference in the survival kinetics of *C. elegans* was observed between the mutant and the WT strains placed in liquid medium (Figure 5B; *p* = 0.777 at 24 h, *p* = 0.660 at 48 h, and *p* = 0.935 at 72 h).

### Cytotoxicity Toward HaCaT Cells *in vitro*

We evaluated the cytotoxicity of *S. lugdunensis* WT and Δ*lytSR* strains toward the human keratinocyte cell line HaCaT. The release of LDH by HaCaT reflecting cell necrosis was identical between the two strains 24 h post infection (36.31 ± 2.46% for WT and 42.41 ± 2.48% for Δ*lytSR*).

### Transcriptional Profiling of the *S. lugdunensis* Δ*lytSR* Strain

To identify genes regulated by the LytSR two-component regulatory system, a transcriptional profile analysis of WT and Δ*lytSR* strains was performed. RNA samples were isolated from both strains at mid-exponential phase (6 h) and late-exponential phase (8 h) of growth, corresponding, respectively, to the maximal expression of *lytSR* and *lrgAB* determined by qRT-PCR analyses as described in the section “*In silico* and Transcription Analyses of the *lytSR locus*”. Overall, deletion of *lytSR* affected the expression of 286 genes compared to the WT strain with a threshold of at least two-fold change (full list in Supplementary Table S2). The expression of 125 genes (80 upregulated and 45 downregulated) and of 177 genes (112 upregulated and 65 downregulated) was affected at 6 and 8 h, respectively. Some genes were upregulated (*n* = 7) or downregulated (*n* = 9) at both times. Transcription of *lrgAB* operon was the most dramatically impacted by the deletion, with *lrgA* and *lrgB* expression at 8 h in the Δ*lytSR* strain decreased by 195- and 162-fold, respectively.

**Figure 6:**
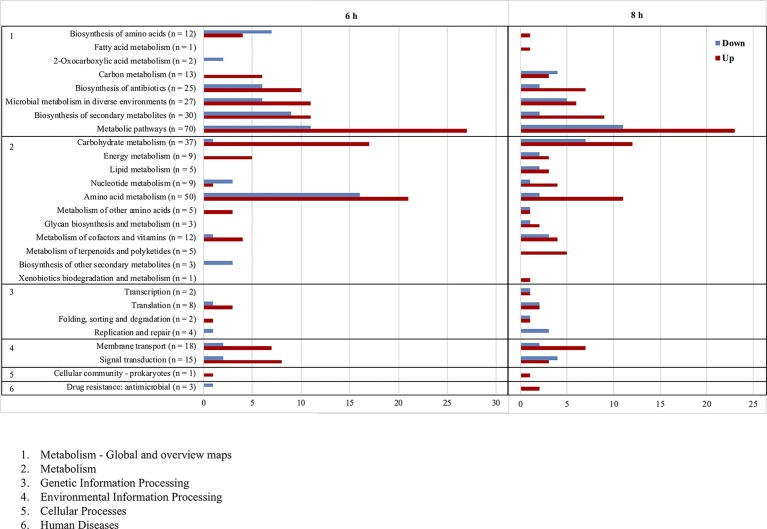
Functional classification according to KEGG pathways of genes significantly upregulated (red) and downregulated (blue) after 6 and 8 h of growth of the ∆*lytSR* strain compared to the WT strain. Numbers in parentheses represent the total number of genes affected within the genome in each functional class.

The main genes upregulated in the Δ*lytSR* strain included those involved in nitrate and nitrite metabolism (*narGHIJ* and *nirBD*), pyruvate metabolism (*cidC*, SLUG_09450, SLUG_02280 to SLUG_02310), amino acid degradation (SLUG_09450, SLUG_09450, SLUG_13970, SLUG_13950, etc.), and nitrogenous bases synthesis (*nrdD*, *rpoE tmk*, etc.; Figure 6). Interestingly, genes encoding known (*fbl*) or putative (*slsB* and *slsC*) surface-anchored proteins as well as iron siderophore uptake proteins (*sstABD*) were upregulated in the Δ*lytSR* strain.

By contrast, genes downregulated in the Δ*lytSR* strain were involved in the pathways of amino acid biosynthesis (*thrBC*, SLUG_15840, SLUG_15710, SLUG_15720, SLUG_15840, SLUG_20810, etc.), nitrogen base degradation (*xpt*, *pyn*, *guaAB*, etc.), and DNA replication pathways (*dnaC* and SLUG_13250; Supplementary Table S2). Other downregulated genes included those encoding autolysins (*atlL* and SLUG_11580), type VII secretion system (*essB*, *esaB,* and *essA*), CAAX amino terminal protease family protein, and two putative secretory antigen precursors (SLUG_07310 and SLUG_21330) homologous to the extracellular immune dominant protein staphylococcal secretory antigen A SsaA.

Moreover, genes encoding transcriptional regulators were upregulated (SLUG_00760 and SLUG_01360) or downregulated (*mgrA* and *sarR*) in the mutant strain.

Results of microarray data were confirmed by qRT-PCR for three genes (*lrgA*, *lrgB*, and *cidA*) among those whose transcription was impacted by the deletion (Table 4).

**Table 4:** Expression of genes regulated by LytSR confirmed by qRT-PCR.

ORF number	Gene	Predicted function	Expression ratio (Δ*lytSR*/WT)	
			Microarray	qRT-PCR
SLUG_05540	*lrgA*	Anti-holin-like protein LrgA	0.005	0.008
SLUG_05530	*lrgB*	LrgA-associated membrane protein LrgB	0.006	0.013
SLUG_04480	*cidA*	Holin-like protein CidA	0.417	0.441

## Discussion

In any biofilm, there is a variable proportion of cells that lyse and release genomic DNA. Because of its adhesive properties, eDNA plays a key role in biofilm matrix (Moormeier and Bayles, 2017). In *S. aureus* and *S. epidermidis*, this process is regulated by the operons *cidABC* and *lrgAB*, under the control of two regulators, CidR and LytSR TCS, respectively (Sadykov and Bayles, 2012). Because LytSR TCS has been identified as a regulator of cell death and lysis in staphylococcal biofilms, we assumed that it could also be involved in the control of these processes in *S. lugdunensis*, which is a particularly virulent species (Frank and Patel, 2007; Pereira et al., 2012; Argemi et al., 2017b). In order to explore the role of this TCS, a *lytSR* knock-out mutant of the WT biofilm-forming strain *S. lugdunensis* DSM 4804 (Freney et al., 1988) was constructed and studied.

First, no difference in growth kinetics or cell viability was observed between mutant and parental strains, indicating that *lytSR* is not essential for bacterial growth in *S. lugdunensis*, as observed for *S. epidermidis* (Zhu et al., 2010). In order to determine whether *lytSR* deletion impacted bacterial autolysis, we performed autolysis assays with Triton X-100, a detergent known to remove the inhibition exerted by lipoteichoic acids on the general bacterial autolytic system. The *S. lugdunensis* Δ*lytSR* strain tended to exhibit the same rate of Triton X-100-induced autolysis as the WT strain, although relatively wide standard deviations were observed at 15- and 30-min time points. This suggests that LytSR was not implicated in induced autolysis for the *S. lugdunensis* DSM 4804 strain. This result is similar to that obtained for the *S. epidermidis* 1457 Δ*lytSR* strain (Zhu et al., 2010). Interestingly, for *S. aureus*, this characteristic appears to be strain-dependent, since the *S. aureus* UAMS-1 *lytS* knock-out mutant did not show increased Triton X-100 induced lysis (Sharma-Kuinkel et al., 2009), whereas the LytSR system plays an important role in the autolysis of the *S. aureus* RN4220 strain (Brunskill and Bayles, 1996a). Thus, the effects of LytSR on autolysis seem to depend on the genetic backgrounds of staphylococcal strains.

In the same way, our work has shown that LytSR was not involved in death induced by antibiotics targeting cell wall synthesis such as penicillin G and glycopeptides. Of note, *lytSR* deletion did not increase sensitivity to the killing effects of glycopeptides toward the *S. lugdunensis* DSM 4804 strain, which is tolerant to glycopeptides (Bourgeois et al., 2007). Time-kill kinetic experiments were conducted on cells growing in early-exponential phase (3 h) as recommended by CLSI (National Committee for Clinical Laboratory Standards, 1999), a time at which *lytSR* and especially *lrgAB* operons were poorly expressed. For *S. aureus*, a *lrgAB* deletion enhanced penicillin-induced killing of cells approaching stationary growth phase, i.e., when *lrgAB* operon is maximally expressed, whereas it did not affect penicillin-induced killing of cells growing in early-exponential phase, when *lrgAB* expression is minimal (Groicher et al., 2000). However, no enhanced killing of penicillin G was observed for *S. lugdunensis* cells reaching stationary growth phase.

Since biofilm formation is one of the main determinants of *S. lugdunensis* pathogenicity (Frank et al., 2008; Argemi et al., 2017b), the effect of *lytSR* deletion on biofilm formation was further investigated. Our results reveal the involvement of LytSR on biofilm production, since the Δ*lytSR* strain produced significantly less biofilm than the WT strain. Moreover, CLSM analyses showed that the mutation disrupted the normal architecture of the biofilm, with a lack of tower structures. This phenotype was only partially restored by *lytSR* complementation. Interestingly, a study has shown that *lrgAB* is specifically expressed within the tower structures during *S. aureus* biofilm formation (Moormeier et al., 2013). Live/dead staining showed a higher rate of red fluorescence inside the mutant biofilm, which is in favor of a decrease in cell viability. Thus, the impaired biofilm formation of the mutant does not seem related to reduced cell lysis, even if to confirm this fact it would be necessary to quantify eDNA levels inside the biofilms. These results are in contradiction with those obtained for other staphylococcal species. For *S. aureus*, a *lytS* mutant and a *lrgAB* mutant produced more adherent biofilm with increased levels of eDNA in the biofilm matrix (Mann et al., 2009; Sharma-Kuinkel et al., 2009). For *S. epidermidis*, a *lytSR* mutant produced more biofilm but contained a significant decrease in the rate of dead cells inside (Zhu et al., 2010). Thus, LytSR is involved in controlling the formation of staphylococcal biofilms, but the pathways of involvement appear to depend on the species.

In order to evaluate the potential involvement of LytSR in the *in vivo* pathogenicity of *S. lugdunensis*, the *C. elegans* nematode was used as a host model organism. This model was chosen because *C. elegans* has already been successfully used for the study of host-pathogen interactions, e.g., for *S. aureus* (Sifri et al., 2003) and *S. lugdunensis* (Gibert et al., 2014). We first used a slow-killing test to observe nematodes’ death after bacterial colonization of intestine as biofilm structures. The mutant strain showed significant attenuation of virulence compared to the WT strain. This observation could be related to its lower ability to form biofilm in the nematode’s intestine (Tan et al., 1999) and *in vitro* (this study). On the contrary, no difference was observed in terms of worm death in liquid killing assay or in LDH release by HaCaT cells. This may be in favor of a similar release of toxins or yet unknown cytoplasmic virulence factors by both strains or be related to a lack of sensitivity of this model.

Another part of this study focused on the impact of *lytSR* on global gene expression by transcriptomic analysis. Two time points were studied, at 6 h of growth equivalent to the mid-exponential phase when the expression of *lytSR* was maximal and at 8 h equivalent to the late-exponential phase when the expression of *lrgAB* was maximal. Of note, it was also found that the *lrgAB* operon transcription of *S. aureus* appeared maximal when cells entered stationary phase (Groicher et al., 2000). Transcription of *lrgAB* was constitutively drastically decreased in the Δ*lytSR* strain, indicating that the operon was activated by LytSR in *S. lugdunensis*. This is consistent with results for *S. aureus* and *S. epidermidis* (Sharma-Kuinkel et al., 2009; Zhu et al., 2010).

In addition, DNA microarray analyses show that the LytSR TCS regulates expression of a wide variety of genes involved in major cellular processes like metabolism of carbohydrates, nucleotides, and amino acids as well as energy metabolism. These results were also observed for other staphylococcal species (Sharma-Kuinkel et al., 2009; Zhu et al., 2010), suggesting that LytSR could affect cell viability and environmental adaptation by regulating the expression of genes controlling bacterial metabolic state. Unlike *S. epidermidis*, genes involved in pyruvate metabolism like the *cidC* gene that encodes a pyruvate:menequinone oxidoreductase were downregulated by LytSR. The higher expression level of *cidC* (2.7) in the *S. lugdunensis* Δ*lytSR* strain might partly explain the higher rate of dead cells observed in Δ*lytSR* biofilm. Indeed, in *S. aureus*, CidC protein plays a major role in bacterial programmed cell death during the stationary phase and in biofilm by converting intracellular pyruvate to acetate (Patton et al., 2005), which leads to cytoplasmic acidification and respiratory inhibition (Thomas et al., 2014). Overflow metabolism and specifically the balance between acetate and acetoin determine the fate of *S. aureus* cells (van den Esker et al., 2017). LytSR could therefore belong to the regulatory networks implicated in the connection between programmed cell death and metabolism in *S. lugdunensis*.

More surprisingly, our results have shown that LytSR was involved in the regulation of genes encoding known or putative virulence and colonization factors (Heilbronner et al., 2011). LytSR seems to downregulate genes encoding surface-anchored proteins including *S. lugdunensis* surface proteins (SlsB and SlsC; Heilbronner et al., 2011) and Fbl, the main fibrinogen-binding protein of *S. lugdunensis* (Marlinghaus et al., 2012). Moreover, the genes *sstABD* encoding putative iron siderophore uptake proteins were shown to be downregulated by LytSR. SstABCD ABC transporter is essential to use catechols and catecholamines as an iron source and therefore to staphylococcal survival and virulence (Beasley et al., 2011).

On the contrary, among genes upregulated in a *lytSR*-dependent manner, are genes encoding the bifunctional autolysin AtlL implicated in *S. lugdunensis* pathogenesis (Gibert et al., 2014; Hussain et al., 2015) as well as SLUG_11580 encoding a putative autolysin. However, our study did not show any involvement of LytSR in autolysis, suggesting that other systems might be involved in the control of autolysins in *S. lugdunensis*. For example, in *S. epidermidis*, *atlE* transcription is regulated by *agr* (Vuong et al., 2003), a system also described in *S. lugdunensis* (Vandenesch et al., 1993) but not yet functionally characterized.

Interestingly, genes encoding the putative type VII secretion system (*essB*, *esaB*, and *essA*) of *S. lugdunensis* were also upregulated by LytSR. These genes have been described by Warne et al. (2016) as homologous to those of *S. aureus*. This secretion system is implicated in the excretion of extracellular proteins across *S. aureus* cell membranes and has been associated with virulence, especially in abscess development (Warne et al., 2016). Several other genes encoding putative virulence factors have been shown to be upregulated by LytSR, like the CAAX amino terminal protease family proteins described as putative membrane-bound metalloproteases (Pei and Grishin, 2001), secretory antigen precursor homologous to the extracellular immune dominant protein SsaA postulated to be involved in *S. epidermidis* biofilm-associated infections (Lang et al., 2000), or the peptide methionine sulfoxide reductase MsrA, which is implicated in oxidative stress tolerance and virulence in *S. aureus* (Singh et al., 2015, 2018).

Of note, apart from *atlL*, no gene involved in *S. lugdunensis* biofilm formation, such as *isdC* (Missineo et al., 2014) or *comEB* (Rajendran et al., 2015), was upregulated by LytSR. The decreased capacity of the Δ*lytSR* strain to form biofilm could therefore be related to the downregulation of *atllL* (Gibert et al., 2014) and s*saA* (Lang et al., 2000; Resch et al., 2005) or be indirectly linked to the altered metabolic state of the cells.

Lastly, LytSR was shown to upregulate (SarA and MarR families) and downregulate (MerR and GntR families) the transcription of several other transcriptional regulators controlling a wide diversity of metabolic processes and virulence (Rodionov, 2007; Jenul and Horswill, 2018). Further studies are necessary to determine whether the involvement of LytSR in the transcription of genes described here is direct or indirect through interaction with other regulators. Especially, electrophoretic gel shift assays could be conducted to determine whether the LytR response regulator binds directly to the promoter regions of its target genes.

## Conclusion

This study is the first to characterize a two-component regulatory system in *S. lugdunensis*, a particularly virulent CoNS. Our results demonstrate that LytSR plays a significant role in the biofilm formation of *S. lugdunensis*, probably in connection with cell death and metabolism. Transcriptional analyses have shed new light on the putative virulence factors of *S. lugdunensis* that could be regulated by LytSR. Furthermore, LytSR appears to play a role in the virulence of *S. lugdunensis*, as evidenced by the *in vivo* model of *C. elegans* infection, but this warrants confirmation in a rat endocarditis model for example. Overall, LytSR appears as a major TCS implicated in *S. lugdunensis* pathogenesis. Further studies of this regulatory system are crucial to investigate how LytSR activation fits into the regulatory networks modulating *S. lugdunensis* virulence.

## Data Availability Statement

Data are available in the Gene Expression Omnibus (GEO), accession number GSE139909.

## Author Contributions

SD, VO, and MP-C designed the study. J-MF, VO, and YZ performed the microarray analyses. TC and OL performed the cytotoxicity assays and *C. elegans* virulence assays. ED, JD, LD, MN, SD, SL, and VO performed all other experiments. SD, VO, and MP-C analyzed the data and wrote the manuscript. All authors have read and approved the final version of the manuscript.

### Conflict of Interest

The authors declare that the research was conducted in the absence of any commercial or financial relationships that could be construed as a potential conflict of interest.

## Footnotes

^1^http://www.kegg.com
