# Supplementary material for: Role of the LytSR Two-Component Regulatory System in Staphylococcus lugdunensis Biofilm Formation and Pathogenesis
Source: Front Microbiol. 2020 Jan 24;11:39. doi: 10.3389/fmicb.2020.00039 (PMC6993578; doi:10.3389/fmicb.2020.00039)
Supplement: Supplementary file 1 [file Data_Sheet_1.PDF]

Supplementary Material

Role of the LytSR two-component regulatory system in *Staphylococcus lugdunensis* biofilm formation and pathogenesis

S. Dahyot*, V. Oxaran, M. Niepceron, E. Dupart, S. Legris, L. Destruel, J. Didi, T. Clamens, O. Lesouhaitier, Y. Zerdoumi, J.M. Flaman, M. Pestel-Caron

*Corresponding author: sandrine.dahyot@chu-rouen.fr

Table S1. Biomass and viability in the biofilm

	**Total Biomass**	**Green fluorescence**	**Red fluorescence**	**Death cell %**
WT	15901.8	15733.6	168.2	1.1
Δ*lytSR*	8014.6	7559.1	455.5	5.7
Δ*lytSR* (pCU1)	3544.3	3007.0	537.3	15.2
Δ*lytSR* (pCU1:*lytSR*)	9984.4	8946.2	1038.2	10.4

Total biomass, live cells stained with SYTO®9 (green fluorescence) and dead cell stained with propidium iodide (red fluorescence) were measured by CLSM and analyzed using IMARIS software.

Table S2. List of genes expressed differentially in Δ*lytSR* strain compared to the wild-type strain.

Complete list of the 80 genes up-regulated at 6 h by the deletion of *lytSR*

Genes involved in	ORF number	Common gene name	Description or predicted function	Fold up-regulation
**<u>1. Metabolism</u>**				
**1.1. Overview**				
Carbon metabolism	SLUG_02280		pyridine nucleotide-disulphide oxidoreductase family protein	3,5
	SLUG_02290		deheydrogenase E1 component family protein	4,1
	SLUG_02300		putative transketolase	3,7
	SLUG_02310		2-oxoacid dehydrogenases acyltransferase family protein	4,1
	SLUG_05050	*gntK*	putative gluconokinase	2,8
	SLUG_15030	*odhB*	dihydrolipoamide succinyltransferase E2 component of 2-ox...	2,6
	SLUG_04660		putative L-serine dehydratase, beta chain	3,1
Biosynthesis of amino acids	SLUG_18900	*argG*	putative argininosuccinate synthase	3,2
	SLUG_18910	*argH*	putative argininosuccinate lyase	3,2
	SLUG_04660		putative L-serine dehydratase, beta chain	3,1
**1.2. Carbohydrate metabolism**				
Glycolysis / Gluconeogenesis	SLUG_02280		pyridine nucleotide-disulphide oxidoreductase family protein	3,5
	SLUG_02290		deheydrogenase E1 component family protein	4,1
	SLUG_02300		putative transketolase	3,7
	SLUG_02310		2-oxoacid dehydrogenases acyltransferase family protein	4,1
	SLUG_07050		PTS system, arbutin-like IIBC component	3,4
	SLUG_03770	*bglA*	6-phospho-beta-glucosidase	3,2
Citrate cycle (TCA cycle)	SLUG_02280		pyridine nucleotide-disulphide oxidoreductase family protein	3,5
	SLUG_02290		deheydrogenase E1 component family protein	4,1
	SLUG_02300		putative transketolase	3,7
	SLUG_02310		2-oxoacid dehydrogenases acyltransferase family protein	4,1
	SLUG_15030	*odhB*	dihydrolipoamide succinyltransferase E2 component of 2-ox...	2,6
Pyruvate metabolism	SLUG_02280		pyridine nucleotide-disulphide oxidoreductase family protein	3,5
	SLUG_02290		deheydrogenase E1 component family protein	4,1
	SLUG_02300		putative transketolase	3,7
	SLUG_02310		2-oxoacid dehydrogenases acyltransferase family protein	4,1
Fructose and mannose metabolism	SLUG_21010	*fruA*	PTS transport system, fructose-specific IIABC component	3,3
Galactose metabolism	SLUG_24140		glycosyl hydrolase family protein	2,9
	SLUG_22490	*tdcB*	putative threonine dehydratase	2,8
Starch and sucrose metabolism	SLUG_24140		glycosyl hydrolase family protein	2,9
	SLUG_22490	*tdcB*	putative threonine dehydratase	2,8
Pentose phosphate pathway	SLUG_05050	*gntK*	putative gluconokinase	2,8
Amino sugar and nucleotide sugar metabolism	SLUG_09130	*glmS / gcaA*	glucosamine--fructose-6-phosphate aminotransferase	2,7
**1.3. Energy metabolism**				
Nitrogen metabolism	SLUG_05940	*nasD / nasBC / nirB*	nitrite reductase large subunit	3,8
	SLUG_05970	*narG*	respiratory nitrate reductase alpha chain	14,8
	SLUG_05980	*narH*	respiratory nitrate reductase beta chain	22,8
	SLUG_05990	*narJ*	respiratory nitrate reductase delta chain	19,0
	SLUG_06000	*narI*	putative nitrate reductase gamma chain	5,2
Porphyrin and chlorophyll metabolism	SLUG_05950	*nasE / nasBD / nirD*	assimilatory nitrite reductase small subunit	5,0
	SLUG_05960		assimilatory nitrite reductase small subunittetrapyrole (corrin/porphyrin) methylase family protein	5,2
Pyrimidine metabolism	SLUG_02690		sodium:solute symporter family protein	2,8
	SLUG_02700		putative nucleoside permease	32,5
	SLUG_02710		putative membrane protein	27,9
	SLUG_02720		PfkB family carbohydrate kinase	28,5
**1.4. Amino acid metabolism**				
Arginine and proline metabolism	SLUG_02450	*odcI*	ornithine decarboxylase	5,1
	SLUG_18900	*argG*	putative argininosuccinate synthase	3,2
	SLUG_18910	*argH*	putative argininosuccinate lyase	3,2
	SLUG_04300		aldehyde dehydrogenase family protein	3,0

Alanine, aspartate and glutamate metabolism	SLUG_22480	*ald2*	alanine dehydrogenase 2	3,7
	SLUG_12190	*ald1*	alanine dehydrogenase 1	5,2
	SLUG_18900	*argG*	putative argininosuccinate synthase	3,2
	SLUG_18910	*argH*	putative argininosuccinate lyase	3,2
	SLUG_04300		aldehyde dehydrogenase family protein	3,0
Amino sugar and nucleotide sugar metabolism	SLUG_09130	*glmS / gcaA*	glucosamine--fructose-6-phosphate aminotransferase [isome...	2,7
Glycine, serine and threonine metabolism	SLUG_02280		pyridine nucleotide-disulphide oxidoreductase family protein	3,5
	SLUG_04660		putative L-serine dehydratase, beta chain	3,1
	SLUG_22570		2-amino-3-ketobutyrate coenzyme A ligase	2,9
Valine, leucine and isoleucine degradation	SLUG_02280		pyridine nucleotide-disulphide oxidoreductase family protein	3,5
Lysine degradation	SLUG_15030	*odhB*	dihydrolipoamide succinyltransferase E2 component of 2-ox...	2,6
Cysteine and methionine metabolism	SLUG_04660		putative L-serine dehydratase, beta chain	3,1
**1.5. Metabolism of other amino acids**				
Glutathione metabolism	SLUG_02450	*odcI*	ornithine decarboxylase	5,1
Taurine and hypotaurine metabolism	SLUG_12190	*ald1*	alanine dehydrogenase 1	5,2
	SLUG_22480	*ald2*	alanine dehydrogenase 2	3,7
**1.6. Metabolism of cofactors and vitamins**				
Porphyrin and chlorophyll metabolism	SLUG_05950	*nasE / nasBD / nirD*	assimilatory nitrite reductase small subunit	5,0
	SLUG_05960		assimilatory nitrite reductase small subunittetrapyrole (corrin/porphyrin) methylase family protein	5,2
Folate biosynthesis	SLUG_20940		conserved hypothetical protein	3,1
	SLUG_20950		putative 6-pyruvoyl tetrahydropterin synthase	3,1
	SLUG_20960		putative radical activating enzyme	3,2
**2. Genetic Information Processing**				
**2.1. Translation**				
Ribosome	SLUG_22670	*rplL*	50S ribosomal protein L7/L12	3,1
	SLUG_13260	*rpsT*	putative 30S ribosomal protein S20	2,7
	SLUG_22610	*rpsG*	30S ribosomal protein S7	2,6
**2.2. Folding, sorting and degradation**				
RNA degradation	SLUG_15900		conserved hypothetical protein	3,2
	SLUG_15910	*miaA*	putative tRNA delta 2-isopentenyl pyrophosphatetransferase	2,8
**3. Environmental Information Processing**				
**3.1. Membrane transport**				
ABC transporters	SLUG_20680	*sstD*	lipoprotein	3,2
	SLUG_20700	*sstB*	FecCD transport family protein	3,1
	SLUG_21680	*mntA*	Manganese ABC transporter ATP-binding protein	4,2
	SLUG_21690	*mntB*	Manganese ABC transporter permease protein	4,9
	SLUG_21700	*mntC*	Manganese ABC transporter extracellular binding protein	3,4
	SLUG_19820		putative solute binding protein	3,7
Phosphotransferase system (PTS)	SLUG_03760		phosphosugar transferase system	4,5
	SLUG_07050		PTS system, arbutin-like IIBC component	3,4
	SLUG_21010	*fruA*	PTS transport system, fructose-specific IIABC component	3,3
**3.2. Signal transduction**				
Two-component system	SLUG_05970	*narG*	respiratory nitrate reductase alpha chain	14,8
	SLUG_05980	*narH*	respiratory nitrate reductase beta chain	22,8
	SLUG_05990	*narJ*	respiratory nitrate reductase delta chain	19,0
	SLUG_06010	*nreA*	hypothetical protein	4,0
	SLUG_06020	*nreB*	putative histidine kinase	5,3
	SLUG_06030	*nreC*	putative response regulator	4,9
	SLUG_06040	*narT*	nitrite transport protein	14,1
**4. Human Diseases**				
**Infectious diseases**				
Staphylococcus aureus infection	SLUG_16350	*fbl*	receptin fbl	2,4
**5. Others**				
Transcription factors	SLUG_00760		GntR family transcriptional regulator	2,7
	SLUG_01360		MerR family transcriptional regulator	3,3
Enzymes	SLUG_01700		putative pyruvate formate-lyase activating enzyme	2,2
Transporters	SLUG_01710	*arsR2*	arsenical resistance operon repressor 2	2,2
	SLUG_02460	*arcD*	arginine/ornithine antiporter	4,6

	SLUG_04650		Transcriptional regulator pfoR	3,2
Transcription factors	SLUG_05040	*gntR*	gluconate operon transcriptional repressor	2,7
	SLUG_05060	*gntP*	putative gluconate permease	3,6
Enzymes, Tansporters	SLUG_06620		putative lipoprotein	2,8
Enzymes, Transporters	SLUG_06630		putative Sec-independent exported protein	2,7
	SLUG_06640		putative membrane protein	2,7
	SLUG_09020		conserved hypothetical protein	2,8
	SLUG_10700		putative membrane protein	2,8
	SLUG_12530		putative GTP-binding protein	3,0
	SLUG_15810		ABC transporter ATP-binding protein	3,2
Transporters, Ion channels	SLUG_15960	*glpF*	putative glycerol uptake facilitator protein	2,5
	SLUG_16500	*epr*	epr protein	2,5
	SLUG_16510	*lytN*	putative cell wall hydrolase	2,5
	SLUG_21140		putative cobalamin synthesis protein	2,2
	SLUG_22300		hypothetical protein	2,9
	SLUG_22380		glycosyltransferase	2,9
	SLUG_22500		amino acid permease	2,5
	SLUG_22600	*fus*	translation elongation factor G	3,1
	SLUG_24130		H+/sugar symporter family protein	4,7
	SLUG_24290		putative short chain dehydrogenase	10,0
	SLUG_24300		major facilitator superfamily protein	11,4
	SLUG_24310		carbohydrate kinase family protein	9,2
	SLUG_24340		putative membrane protein	2,1
	SLUG_24350		putative membrane protein	2,1

Complete list of the 112 genes up-regulated at 8 h by the deletion of *lytSR*

Genes involved in	ORF number	Common gene name	Description or predicted function	Fold up-regulation
**1. Metabolism**				
**Overview**				
Fatty acid metabolism	SLUG_02150		short chain dehydrogenase	3,5
Carbon metabolism	SLUG_01310		PfkB family protein	2,5
	SLUG_01320		KDPG and KHG aldolase family protein	2,2
Biosynthesis of amino acids	SLUG_03580		putative peptidase	2,3
	SLUG_05160		putative peptidase	2,1
2-Oxocarboxylic acid metabolism	SLUG_05160		putative peptidase	2,1
**Carbohydrate metabolism**				
Propanoate metabolism	SLUG_09450		aldehyde dehydrogenase family protein	2,7
Pyruvate metabolism	SLUG_04500	*cidC*	thiamine pyrophosphate enzyme	2,7
	SLUG_09450		aldehyde dehydrogenase family protein	2,7
Pentose phosphate pathway	SLUG_01310		PfkB family protein	2,5
	SLUG_01320		KDPG and KHG aldolase family protein	2,2
Pentose and glucuronate interconversions	SLUG_09450		aldehyde dehydrogenase family protein	2,7
Glycolysis / Gluconeogenesis	SLUG_09450		aldehyde dehydrogenase family protein	2,7
Ascorbate and aldarate metabolism	SLUG_09450		aldehyde dehydrogenase family protein	2,7
Glyoxylate and dicarboxylate metabolism	SLUG_15650	*katA*	catalase	2,4
	SLUG_01320		KDPG and KHG aldolase family protein	2,2
Butanoate metabolism	SLUG_05410		zinc dependent dehydrogenase	2,1
**Energy metabolism**				
Nitrogen metabolism	SLUG_19440		putative dioxygenase	4,1
	SLUG_05970	*narG*	nitrate reductase alpha chain	2,3
	SLUG_05980	*narH*	nitrate reductase beta chain	2,4
	SLUG_05990	*narJ*	respiratory nitrate reductase delta chain	2,1
**Lipid metabolism**				
Fatty acid biosynthesis	SLUG_02150		short chain dehydrogenase	3,5
Biosynthesis of unsaturated fatty acids	SLUG_02150		short chain dehydrogenase	3,5
Fatty acid degradation	SLUG_09450		aldehyde dehydrogenase family protein	2,7
Glycerolipid metabolism	SLUG_09450		aldehyde dehydrogenase family protein	2,7
**Nucleotide metabolism**				
Purine metabolism	SLUG_03600	*nrdD*	anaerobic ribonucleoside-triphosphate reductase	3,5
	SLUG_09390	*rpoE*	DNA-directed RNA polymerase delta subunit	3,3
Pyrimidine metabolism	SLUG_18570		conserved hypothetical protein	2,1
	SLUG_03600	*nrdD*	anaerobic ribonucleoside-triphosphate reductase	3,5
	SLUG_09390	*rpoE*	DNA-directed RNA polymerase delta subunit	3,3
**Amino acid metabolism**	SLUG_23260	*tmk*	putative thymidylate kinase	2,3
Histidine metabolism				
Lysine degradation	SLUG_09450		aldehyde dehydrogenase family protein	2,7
Valine, leucine and isoleucine degradation	SLUG_09450		aldehyde dehydrogenase family protein	2,7
	SLUG_09450		aldehyde dehydrogenase family protein	2,7
	SLUG_13950	*bfmBAA (bfmB1A)*	2-oxoisovalerate dehydrogenase alpha subunit	2,2
	SLUG_13960	*bfmBAB (bfmB1B)*	2-oxoisovalerate dehydrogenase beta subunit	2,3
Tryptophan metabolism	SLUG_13970	*bfmB (bfmB2, bfmBB)*	lipoamide acyltransferase component of branched-chain alp...	2,6
	SLUG_09450		aldehyde dehydrogenase family protein	2,7
Arginine and proline metabolism	SLUG_15650	*katA*	catalase	2,4
	SLUG_09450		aldehyde dehydrogenase family protein	2,7
	SLUG_03140	*arcA*	arginine deiminase	2,4
Lysine biosynthesis	SLUG_05160		putative peptidase	2,1
**Metabolism of other amino acids**	SLUG_03580		putative peptidase	2,3
beta-Alanine metabolism				
**Glycan biosynthesis and metabolism**	SLUG_09450		aldehyde dehydrogenase family protein	2,7
Peptidoglycan biosynthesis				
	SLUG_17190	*pbpA*	penicillin-binding protein 1	2,4
**Metabolism of cofactors and vitamins**	SLUG_17180	*mraY*	phospho-N-acetylmuramoyl-pentapeptide-transferase	2,1
Biotin metabolism				
Pantothenate and CoA biosynthesis	SLUG_02150		short chain dehydrogenase	3,5
Nicotinate and nicotinamide metabolism	SLUG_01080		putative ketopantoate reductase	2,8
**Metabolism of terpenoids and polyketides**	SLUG_18560		conserved hypothetical protein	2,4
Carotenoid biosynthesis				
	SLUG_01820		putative membrane protein	2,8
	SLUG_01830		putative phytoene dehydrogenase related protein	3,3
	SLUG_01840		putative glycosyl transferase	4,0
	SLUG_01850	*crtM*	squalene desaturase	3,5
Limonene and pinene degradation	SLUG_01860	*crtN*	squalene synthase	2,8
**Xenobiotics biodegradation and metabolism**	SLUG_09450		aldehyde dehydrogenase family protein	2,7
Chloroalkane and chloroalkene degradation				
	SLUG_09450		aldehyde dehydrogenase family protein	2,7
**2. Genetic Information Processing**				
**Transcription**				
RNA polymerase				
**Translation**	SLUG_09390	*rpoE*	DNA-directed RNA polymerase delta subunit	3,3
Aminoacyl-tRNA biosynthesis				
	SLUG_01370	*thrZ*	threonyl-tRNA synthetase	2,9
**Folding, sorting and degradation**	SLUG_15630		30S ribosomal protein S14	2,4
RNA degradation				

		SLUG_15900		conserved hypothetical protein	5,3
**3. Environmental Information Processing**					
**Membrane transport**					
	ABC transporters				
		SLUG_05420	*opuCA*	putative glycine betaine/carnitine/choline transport ATP-binding protein	3,1
		SLUG_05430	*opuCB*	putative glycine betaine/carnitine/choline transport system permease protein	
		SLUG_05440	*opuCC*	putative glycinebetaine/carnitine/choline-binding lipoprotein	2,9
		SLUG_07410		BioY family protein	2,6
		SLUG_17680		ABC transporter extracellular binding protein	2,5
		SLUG_20830		ABC transporter permease protein	2,5
		SLUG_20840		ABC transporter ATP-binding protein	2,4
**Signal transduction**					
	Two-component system				
		SLUG_05970	*narG*	nitrate reductase alpha chain	2,3
		SLUG_05980	*narH*	nitrate reductase beta chain	2,4
		SLUG_05990	*narJ*	respiratory nitrate reductase delta chain	2,1
**4. Human Diseases**					
**Infectious diseases**					
	Staphylococcus aureu s infection				
**Drug resistance**		SLUG_16350	*fbl*	receptin fbl	3,0
	beta-Lactam resistance				
		SLUG_17190	*pbpA*	penicillin-binding protein 1	2,4
**5. Others**					
		SLUG_01360		MerR family transcriptional regulator	2,4
		SLUG_01380		OxyL-like protein	3,8
	Enzymes	SLUG_01670		putative reductase	2,0
		SLUG_01700		putative pyruvate formate-lyase activating enzyme	3,3
		SLUG_02070		conserved hypothetical protein	2,9
		SLUG_02090		putative membrane protein	2,3
		SLUG_02100		hypothetical membrane protein	2,2
		SLUG_02140		putative N-acetyltransferase	2,8
		SLUG_02250		putative membrane protein	3,3
		SLUG_02630		hypothetical protein	2,7
		SLUG_02970		conserved hypothetical protein	2,1
		SLUG_03440		conserved membrane protein	2,9
	Enzymes	SLUG_03550		sulfite exporter TauE/SafE family protein	2,3
		SLUG_03610	*nrdG*	putative anaerobic ribonucleotide reductase activating pr...	3,1
		SLUG_03620		hypothetical membrane protein	2,4
		SLUG_03850	*slsc*	putative LPXTG cell wall-anchored protein	2,6
		SLUG_03860		putative membrane protein	3,1
		SLUG_04020		conserved hypothetical protein	3,0
		SLUG_04150		amino acid permease family protein	2,8
		SLUG_04170		conserved hypothetical protein	2,7
		SLUG_04330		conserved hypothetical protein	2,5
		SLUG_04340		CorA-like Mg2+ transporter protein	4,6
		SLUG_04760	*slsB*	putative LPXTG cell wall-anchored protein	2,7
		SLUG_04790		acetyltransferase (GNAT) family protein	2,2
		SLUG_05860		putative membrane protein	2,5
		SLUG_06100		conserved hypothetical protein	2,4
		SLUG_06700	*lldP2*	putative L-lactate permease 2	2,8
	Chaperones and folding catalysts	SLUG_07000		putative short chain dehydrogenase	2,2
		SLUG_07360	*clpL*	putative ATP-dependent protease ATP-binding subunit ClpL	3,2
		SLUG_08930		putative membrane protein	2,6
		SLUG_08950	*asp23*	alkaline shock protein 23	2,6
		SLUG_09330		putative peptidase	2,5
		SLUG_09340		hypothetical protein	3,7
		SLUG_09360		hypothetical protein	2,6
		SLUG_09380		putative acetyltransferase	3,2
	Enzymes, Peptidases	SLUG_10700		putative membrane protein	2,4
		SLUG_10930		ThiJ/PfpI family protein	3,5
		SLUG_11210		putative membrane protein	2,1
		SLUG_11490		aldo/keto reductase family protein	2,3
		SLUG_11760		putative exported protein / General stress protein-like protein	2,2
		SLUG_11890		putative exported protein	2,5
	Enzymes, Peptidases, DNA repair and recombination proteins	SLUG_11900		putative membrane protein	2,9
		SLUG_15590	*dinR (lexA)*	DNA damage-inducible repressor	2,0
	Enzymes, Prenyltransferases, Transfer RNA biogenesis	SLUG_15780		hypothetical uncharacterized protein	2,4
		SLUG_15910	*miaA*	putative tRNA delta 2-isopentenyl pyrophosphatetransferase	5,1
		SLUG_16040		conserved hypothetical protein	2,4
		SLUG_16620		hypothetical protein	2,4
		SLUG_17240		acetyltransferase (GNAT) family protein	3,5
		SLUG_17920		hypothetical protein	3,3
	Enzymes	SLUG_18280		hypothetical protein	2,5
		SLUG_18550		putative RNA pseudouridylate synthase	2,5
		SLUG_19450		putative CBS domains-containing hemolysin	3,1
		SLUG_19810		conserved hypothetical protein	3,1
		SLUG_19930		OsmC-like protein	2,3

Transcription factors	SLUG_21030		DeoR ramily regulatory protein	3,2
	SLUG_21170		putative exported protein	2,6
	SLUG_21200		conserved hypothetical protein (pseudogene)	3,9
	SLUG_21210		hypothetical protein	2,2
	SLUG_21320		putative membrane protein	2,2
Transporters	SLUG_21745		Na(+) H(+) antiporter subunit C	2,3
Transporters	SLUG_21760		Na(+) H(+) antiporter subunit B	2,5
Transporters	SLUG_21770		Na(+) H(+) antiporter subunit A	2,3
	SLUG_21860		putative exported protein	3,5
	SLUG_22040		aldo/keto reductase family protein	2,5
	SLUG_22170		hypothetical protein	2,6
	SLUG_22180		hypothetical protein	3,6
	SLUG_22210		putative amino acid permease	2,8
	SLUG_22290	*scdA*	cell wall metabolism protein	3,0
Transporters	SLUG_22310	*proP*	putative proline/betaine transporter	3,5
	SLUG_23710		hypothetical protein	2,6
	SLUG_23810		putative membrane protein	3,1

Complete list of the 45 genes down-regulated at 6 h by the deletion of *lytSR*

Genes involved in		ORF number	Common gene name	Description or predicted function	Fold down-regulation
**<u>1. Metabolism</u>**					
**1.1. Overview**					
	Biosynthesis of amino acids	SLUG_15690	*thrB*	homoserine kinase	7,1
		SLUG_15700	*thrC*	threonine synthase	7,5
		SLUG_15710		putative homoserine dehydrogenase	6,3
		SLUG_15720		putative aspartate kinase	3,4
		SLUG_15840		conserved hypothetical protein	3,4
		SLUG_20810		putative aminotransferase	2,7
		SLUG_14830	*lysC*	aspartokinase II	2,6
	2-Oxocarboxylic acid metabolism	SLUG_15720		putative aspartate kinase	3,4
		SLUG_14830	*lysC*	aspartokinase II	2,6
**1.2. Carbohydrate metabolism**					
	Amino sugar and nucleotide sugar metabolism	SLUG_09120		putative phosphoglucosamine mutase	2,3
**1.3. Nucleotide metabolism**					
	Purine metabolism	SLUG_23660	*guaA*	putative GMP synthase	3,0
		SLUG_23670	*guaB*	putative inosine-5'-monophosphate dehydrogenase	3,0
		SLUG_23690	*xpt*	putative xanthine phosphoribosyltransferase	3,3
**1.4. Amino acid metabolism**					
	Glycine, serine and threonine metabolism	SLUG_15690	*thrB*	homoserine kinase	7,1
		SLUG_15700	*thrC*	threonine synthase	7,5
		SLUG_15710		putative homoserine dehydrogenase	6,3
		SLUG_15720		putative aspartate kinase	3,4
		SLUG_15840		conserved hypothetical protein	3,4
		SLUG_14830	*lysC*	aspartokinase II	2,6
	Cysteine and methionine metabolism	SLUG_15710		putative homoserine dehydrogenase	6,3
		SLUG_15720		putative aspartate kinase	3,4
		SLUG_14830	*lysC*	aspartokinase II	2,6
	Lysine biosynthesis	SLUG_15710		putative homoserine dehydrogenase	6,3
		SLUG_15720		putative aspartate kinase	3,4
		SLUG_14830	*lysC*	aspartokinase II	2,6
	Histidine metabolism	SLUG_20810		putative aminotransferase	2,7
	Tyrosine metabolism	SLUG_20810		putative aminotransferase	2,7
	Phenylalanine metabolism	SLUG_20810		putative aminotransferase	2,7
	Phenylalanine, tyrosine and tryptophan biosynthesis	SLUG_20810		putative aminotransferase	2,7
**1.5. Metabolism of cofactors and vitamins**					
	Vitamin B6 metabolism	SLUG_15690	*thrB*	homoserine kinase	7,1
		SLUG_15700	*thrC*	threonine synthase	7,5
**1.6. Biosynthesis of other secondary metabolites**					
	Novobiocin biosynthesis	SLUG_20810		putative aminotransferase	2,7
**1.7. Xenobiotics biodegradation and metabolism**					
**<u>2. Genetic Information Processing</u>**					
**2.1. Translation**					
	Aminoacyl-tRNA biosynthesis	SLUG_21940	*argS*	putative arginyl-tRNA synthetase	2,3
**2.2. Replication and repair**					
	DNA replication	SLUG_00150	*dnaC*	DnaB-like helicase	2,4
**<u>3. Environmental Information Processing</u>**					
**3.1. Membrane transport**					
	ABC transporters	SLUG_06780		ABC transporter ATP-binding protein	6,0
		SLUG_23470		putative transport system membrane protein	2,8
**3.2. Signal transduction**					
	Two-component system	SLUG_05540	*lrgA*	holin-like protein	3,3
**<u>4. Human Diseases</u>**					
**Infectious diseases**					
	Staphylococcus aureus infection	SLUG_19300	*dltD*	putative lipoteichoic acid biosynthesis protein	2,1
**<u>5. Others</u>**					
		SLUG_01590		conserved hypothetical membrane protein	3,0
		SLUG_02030		MarR family transcriptional regulator	2,5
		SLUG_02040	*isaB*	immunodominant antigen B	2,5
		SLUG_03650		conserved hypothetical protein	2,4
		SLUG_05380		putative transport protein	2,2
		SLUG_05490		putative membrane protein	2,6
	Transporters	SLUG_05570		putative transport protein	2,7
		SLUG_05600		hypothetical protein	2,7
	Transporters	SLUG_06820	*tcaB*	teicoplanin resistance associated membrane protein	2,1
		SLUG_06950		conserved hypothetical protein	2,6

	SLUG_07350	*sarR*	staphylococcal accessory regulator A homologue	2,7
Enzymes, Peptidases	SLUG_10100		putative acetyltransferase	2,1
Transfer RNA biogenesis , Prokaryotic Type Ribosome biogenesis	SLUG_10110		putative glycoprotease	2,1
	SLUG_10160		conserved hypothetical protein	3,0
	SLUG_10390		hypothetical protein	2,9
	SLUG_10880		conserved hypothetical protein	3,5
	SLUG_10890		putative aminopeptidase	4,2
	SLUG_10900		conserved hypothetical protein	4,2
Enzymes, Peptidases	SLUG_13820		putative peptidase	2,5
	SLUG_15660		amino acid permease	2,8
	SLUG_15680		haloacid dehalogenase-like hydrolase	2,2
Enzymes	SLUG_18190	*atl*	bifunctional autolysin precursor	2,2
Enzymes, Peptidases	SLUG_18620	*pepB*	putative oligopeptidase	3,2
	SLUG_21450		conserved hypothetical protein	2,5
	SLUG_21490		putative membrane protein	2,5
	SLUG_23410		putative membrane protein	2,5
Transporters	SLUG_23680	*pbuX*	putative xanthine permease	4,3
	SLUG_23820		hypothetical protein	3,1

Complete list of the 65 genes down-regulated at 8 h by the deletion of *lytSR*

Genes involved in		ORF number	Common gene name	Description or predicted function	Fold down-regulation
<u>**1. Metabolism**</u>					
**1.1. Overview**					
	Carbon metabolism	SLUG_21640		putative D-isomer specific 2-hydroxyacid dehydrogenase	3,3
		SLUG_13750	*gcvT*	putative aminomethyltransferase	2,5
		SLUG_22130	*pta*	putative phosphate acetyltransferase	2,2
		SLUG_11980	*fhs*	formate--tetrahydrofolate ligase	2,1
**1.2. Carbohydrate metabolism**					
	Glyoxylate and dicarboxylate metabolism	SLUG_21640		putative D-isomer specific 2-hydroxyacid dehydrogenase	3,3
	Pentose phosphate pathway	SLUG_09270	*deoC1*	deoxyribose-phosphate aldolase	3,0
	Glyoxylate and dicarboxylate metabolism	SLUG_05720		putative glycerate kinase	2,6
		SLUG_04230		D-isomer specific 2-hydroxyacid dehydrogenase	2,3
	Pyruvate metabolism	SLUG_22130	*pta*	putative phosphate acetyltransferase	2,2
	Propanoate metabolism	SLUG_22130	*pta*	putative phosphate acetyltransferase	2,2
**1.3. Energy metabolism**					
	Methane metabolism	SLUG_21640		putative D-isomer specific 2-hydroxyacid dehydrogenase	3,3
		SLUG_22130	*pta*	putative phosphate acetyltransferase	2,2
**1.4. Lipid metabolism**					
	Glycerolipid metabolism	SLUG_05720		putative glycerate kinase	2,6
	Secondary bile acid biosynthesis	SLUG_02810		putative choloylglycine hydrolase	2,2
**1.5. Nucleotide metabolism**					
	Purine metabolism	SLUG_22650	*rpoB*	DNA-directed RNA polymerase beta chain protein	2,3
		SLUG_13250		DNA polymerase III delta subunit	2,3
	Pyrimidine metabolism	SLUG_22650	*rpoB*	DNA-directed RNA polymerase beta chain protein	2,3
		SLUG_13250		DNA polymerase III delta subunit	2,3
		SLUG_09280	*pyn / pdp*	putative pyrimidine-nucleoside phosphorylase	2,2
**1.6. Amino acid metabolism**					
	Glycine, serine and threonine metabolism	SLUG_05720		putative glycerate kinase	2,6
		SLUG_13750	*gcvT*	putative aminomethyltransferase	2,5
**1.7. Metabolism of other amino acids**					
	Taurine and hypotaurine metabolism	SLUG_22130	*pta*	putative phosphate acetyltransferase	2,2
**1.8. Metabolism of cofactors and vitamins**					
	One carbon pool by folate	SLUG_13750	*gcvT*	putative aminomethyltransferase	2,5
		SLUG_11980	*fhs*	formate--tetrahydrofolate ligase	2,1
<u>**2. Genetic Information Processing**</u>					
**2.1. Transcription**					
	RNA polymerase	SLUG_22650	*rpoB*	DNA-directed RNA polymerase beta chain protein	2,3
**2.2. Translation**					
	Aminoacyl-tRNA biosynthesis	SLUG_22790	*gltX*	putative glutamyl-tRNA synthetase	2,3
	Ribosome	SLUG_16790	*rpmB*	50S ribosomal protein L28	2,0
**2.3. Replication and repair**					
	DNA replication	SLUG_13250		DNA polymerase III delta subunit	2,3
	Mismatch repair	SLUG_13250		DNA polymerase III delta subunit	2,3
	Homologous recombination	SLUG_13250		DNA polymerase III delta subunit	2,3
<u>**3. Environmental Information Processing**</u>					
**3.1. Membrane transport**					
	ABC transporters	SLUG_21680	*mntA*	ABC transporter ATP-binding protein	3,2
		SLUG_21690	*mntB*	ABC transporter extracellular binding protein	2,7
**3.2. Signal transduction**					
	Two-component system	SLUG_05540	*lrgA*	holin-like protein	195,0
		SLUG_05530	*lrgB*	holin-like protein	162,1
		SLUG_00860	*kdpA (SCCmec)*	potassium-transporting ATPase A chain	2,2
		SLUG_00880	*kdpC (SCCmec)*	potassium-transporting ATPase C chain	2,1
<u>**4. Others**</u>					

	SLUG_00670		hypothetical lipoprotein	2,0
	SLUG_00680		hypothetical lipoprotein x3	2,2
	SLUG_00930	*IsdJ*	putative LPXTG cell wall-anchored NEAT domain protein	2,2
	SLUG_01060		acetyltransferase family protein	2,3
Enzymes	SLUG_01550	*msrA3*	hypothetical protein	2,2
	SLUG_01790		conserved hypothetical protein	2,1
	SLUG_02750		CAAX amino terminal protease family protein	2,1
	SLUG_02760		CAAX amino terminal protease family protein	2,3
Transporters	SLUG_04480	*cidA*	hypothetical protein	2,4
Enzymes, Peptidases	SLUG_04810		peptidase M48 family protein	2,4
Transporters	SLUG_04970		putative transporter protein	2,1
	SLUG_05140		hypothetical protein	2,2
	SLUG_05380		putative transport protein	2,4
	SLUG_05490		putative membrane protein	5,5
	SLUG_05500		Putative EsaC protein analog	2,5
	SLUG_07310		putative exported protein / Secretory antigen precursor SsaA	2,4
Translation factors	SLUG_10130		ABC transporter ATP-binding protein	2,1
	SLUG_10330		putative membrane protein	2,6
	SLUG_10890		putative aminopeptidase	2,2
	SLUG_10900		conserved hypothetical protein	2,1
	SLUG_11580		putative exported protein / Autolysin	3,2
	SLUG_11670		conserved hypothetical protein	2,3
	SLUG_11710		conserved hypothetical protein	2,3
Enzymes	SLUG_12150		putative thiol peroxidase	2,4
Transcription factors	SLUG_12910		conserved hypothetical protein	2,1
	SLUG_13400		PhoH-like protein	2,3
Enzymes, Peptidases	SLUG_13820		putative peptidase	2,6
	SLUG_15070		putative membrane protein	2,2
Enzymes	SLUG_15380	*msrA2*	peptide methionine sulfoxide reductase I	2,0
	SLUG_16100	*cvfA*	putative exported protein / Hydrolase (HAD superfamily)	2,4
	SLUG_17590		conserved hypothetical protein	2,4
Enzymes, Peptidases	SLUG_18620	*pepB*	putative oligopeptidase	2,2
	SLUG_19660	*essB*	Putative secretion system component EssB/YukC	2,3
	SLUG_19670	*esaB*	Putative secretion accessory protein EsaB/YukD	2,4
	SLUG_19680	*essA*	Putative secretion system component EssA	2,5
	SLUG_20070		putative lysin decarboxylase family protein	2,2
	SLUG_20440		putative exported protein / putative secretory antigen precursor	3,5
	SLUG_21050		putative exported protein	2,5
	SLUG_21150	*mgrA*	MarR family regulatory protein	2,9
	SLUG_21330		putative exported protein / Secretory antigen precursor SsaA	2,8
	SLUG_21450		conserved hypothetical protein	2,4
	SLUG_21630		conserved hypothetical protein	4,4
Enzymes	SLUG_22140		conserved hypothetical protein	2,6
Transporters	SLUG_23680	*pbuX*	putative xanthine permease	2,2
	SLUG_23750		conserved hypothetical protein	2,7
	SLUG_24160		hypothetical exported protein	3,7
